# Preferential Activation of the Hedgehog Pathway by Epigenetic Modulations in HPV Negative HNSCC Identified with Meta-Pathway Analysis

**DOI:** 10.1371/journal.pone.0078127

**Published:** 2013-11-04

**Authors:** Elana J. Fertig, Ana Markovic, Ludmila V. Danilova, Daria A. Gaykalova, Leslie Cope, Christine H. Chung, Michael F. Ochs, Joseph A. Califano

**Affiliations:** 1 Department of Oncology, Sidney Kimmel Comprehensive Cancer Center, Johns Hopkins University, Baltimore, Maryland, United States of America; 2 Department of Hematopoietic Malignancies, Hellen Diller Family Comprehensive Cancer Center, University of California San Francisco, San Francisco, California, United States of America; 3 Department of Otolaryngology-Head and Neck Surgery, Johns Hopkins Medical Institutions, Baltimore, Maryland, United States of America; 4 Department of Health Science Informatics, School of Medicine, Johns Hopkins University, Baltimore, Maryland, United States of America; 5 Milton J. Dance Head and Neck Center, Greater Baltimore Medical Center, Baltimore, Maryland, United States of America; Karolinska Institutet, Sweden

## Abstract

Head and neck squamous cell carcinoma (HNSCC) is largely divided into two groups based on their etiology, human papillomavirus (HPV)-positive and –negative. Global DNA methylation changes are known to drive oncogene and tumor suppressor expression in primary HNSCC of both types. However, significant heterogeneity in DNA methylation within the groups results in different transcriptional profiles and clinical outcomes. We applied a meta-pathway analysis to link gene expression changes to DNA methylation in distinguishing HNSCC subtypes. This approach isolated specific epigenetic changes controlling expression in HPV− HNSCC that distinguish it from HPV+ HNSCC. Analysis of genes identified Hedgehog pathway activation specific to HPV− HNSCC. We confirmed that *GLI1*, the primary Hedgehog target, showed higher expression in tumors compared to normal samples with HPV− tumors having the highest *GLI1* expression, suggesting that increased expression of *GLI1* is a potential driver in HPV− HNSCC. Our algorithm for integration of DNA methylation and gene expression can infer biologically significant molecular pathways that may be exploited as therapeutics targets. Our results suggest that therapeutics targeting the Hedgehog pathway may be of benefit in HPV− HNSCC. Similar integrative analysis of high-throughput coupled DNA methylation and expression datasets may yield novel insights into deregulated pathways in other cancers.

## Introduction

Head and neck squamous cell carcinoma (HNSCC) is heterogeneous, arising in multiple sites within the head and neck region with diverse risk factors, including tobacco and alcohol use, and human papillomavirus (HPV) infection [1]. Nonsmoking patients with advanced stage HPV-related (HPV-positive) tumors have a significantly better outcome compared to HPV-negative patients [2]. Recent clinical trials have focused on exploiting the favorable prognosis for HPV-positive tumors by de-intensification of therapy in order to decrease unnecessary treatment-related toxicities, such as the use of cetuximab, a monoclonal antibody against the epidermal growth factor receptor (EGFR) in place of cytotoxic systemic agents for locally advanced disease. However, advanced stage HPV-negative HNSCC continues to have a dismal prognosis, and development of novel targeted therapies through molecular characterization and specific targeting of deregulated pathways would greatly benefit this group of patients.

While the concept of biologically driven therapy targeting deregulated pathways is promising, the biologic complexities of cancer [3] render any single data modality insufficient to identify oncogenic drivers. Such oncogenic driver identification has often been limited to inferring unambiguous genetic alterations with analysis of DNA copy number variation or exon mutation. Nonetheless, epigenetic re-activation of oncogenes through global DNA hypomethylation and inactivation of tumor suppressor gene pathways have been observed in HNSCC [4]. Such changes in DNA methylation have also been found to distinguish HPV-positive from HPV-negative HNSCC [5]. Although such global changes have been inferred in HNSCC, their link to expression and functional changes are currently limited. Notably, the identification of epigenetic drivers and integration of epigenetic data with expression data are hindered by the large volume and heterogeneous nature of epigenetic alteration in HNSCC, and more generally in primary solid tumors.

To define collections of genes active in subtypes in HNSCC (meta-pathways), we applied a cross-platform analysis to integrate DNA methylation and expression arrays of 44 HNSCC and 25 normal samples, exploiting epigenetic re-activation of oncogenes through global DNA hypomethylation in HNSCC [4]. The resulting analysis identified global patterns in gene expression driven by methylation changes in specific samples from the high-throughput data, without encoding information about the clinical phenotypes of samples. Nonetheless, these patterns were associated with changes linked to tumor samples, HPV status, gender, and tumor subsite. The group of genes associated with inferred differences between sample groups together define “meta-pathways” associated with these phenotypes, which were linked to canonical pathways associated with human cancers. As a result, the algorithm inferred pathways that reflect the biology of HNSCC [1] and also identified novel coordinated DNA methylation and expression changes in *GLI1* expression in a subset of HPV-negative HNSCC tumors.

## Methods

### Sample Preparation and Generation of Array Data

76 surgical tumor samples from HNSCC and 40 normal samples from uvulopalatopharyngoplasty (UPPP) were obtained from the Head and Neck tissue bank at Johns Hopkins, acquired under Hopkins Internal Review Board approved research protocols. Samples were treated using standard protocols for recovery of DNA and RNA. 44 HNSCC and 25 UPPP samples were run on Affymetrix HuEx1.0 GeneChips and Illumina Infinium HumanMethylation27 BeadChips. All arrays were run according to manufacturer protocols. The other 32 surgical tumors from HNSCC and 15 normal UPPP samples were reserved for independent validation of findings from the discovery cohort. Table 1 describes the clinical attributes of the samples from both the discovery and the validation cohort.

**Table 1:** Clinical attributes of samples Summary of clinical features of samples in discovery, validation, and TCGA sample cohorts.

	Discovery			Test			TCGA		
	Normal	HPV+	HPV−	Normal	HPV+	HPV−	Normal	HPV+	HPV−
	(n = 25)	(n = 13)	(n = 31)	(n = 15)	(n = 11)	(n = 21)	(n = 50)	(n = 35)	(n = 244)
**Gender**									
Female	16	2	10	10	1	7	12	4	72
Male	9	11	21	5	10	14	38	31	172
**Race**									
Caucasian	14	12	28	9	9	18	42	33	209
African American	11	0	3	6	1	3	6	2	24
Other	0	1	0	0	1	1	2	0	11
**Smoking**									
Yes	3	8	19	7	8	14	41	25	195
No	22	4	8	8	2	6	9	10	41
Unknown	0	1	4	0	1	1	0	0	8
**Alcohol**									
Yes	9	9	16	0	5	12	36	29	159
No	16	2	10	15	5	8	13	5	80
Unknown	0	2	5	0	1	1	1	1	5
**Tumor Site**									
Oral Cavity		0	10		1	10		12	160
Oropharynx		11	6		10	4		21	12
Larynx		2	11		0	6		1	71
Hypopharynx		0	4		0	1		1	1
**T stage**									
1		4	9		1	2		3	17
2		7	5		4	4		10	63
3		1	6		1	2		2	54
4		1	10		0	0		0	0
4A		0	1		2	3		9	86
4B		0	0		0	0		0	1
X		0	0		0	0		7	20
Unknown		0	0		3	10		4	3
**N stage**									
0		1	13		3	4		11	80
1		1	4		1	3		3	29
2		0	1		0	0		1	5
2A		4	2		0	0		1	1
2B		5	9		3	1		5	48
2C		2	2		1	3		0	29
3		0	0		0	0		0	4
X		0	0		0	0		10	44
Unknown		0	0		3	10		4	4

Affymetrix HuEx1.0 GeneChips gene expression data was normalized with RMA with the Bioconductor oligo package [6]. Gene level summaries were obtained by averaging normalized, transcript-level expression estimates for core probes annotated to that gene. For DNA methylation, bisulfite treated samples were hybridized to the arrays, where a pair of probes correspond to each CpG loci. One of these probes (the **M** probe) corresponds to the reference genome sequence, and targets methylated DNA, while the other (denoted **U** for unmethylated) reflects the C-to-T conversion that bisulfite treatment induces in DNA not protected by methylation. We converted these values to locus-specific methylation according to 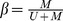, with custom R scripts that filtered probes with less than three CpGs. Gene level summaries for DNA methylation represented the maximum β value in all probes annotated to a gene. Gene annotations for the methylation array were obtained from the Bioconductor IlluminaHumanMethylation27 k.db package and for the expression array from ASAP [7].

All high throughput data sets are available in GEO. The data is in superSeries GSE33232, with individual data sets available: Affymetrix Expression Data, GEO33205 and Illumina Methylation Data, GEO33202. All R code used to generate the results is included in the zip archive in Methods S1.

### Meta-pathway Analysis of Integrated Gene Expression and DNA Methylation Data

Meta-pathway analyses were performed using a Bayesian Markov chain Monte Carlo (MCMC) non-negative matrix factorization algorithm described in [8] and implemented in the Bioconductor package CoGAPS (Coordinated Gene Activity in Pattern Sets; [9]). This algorithm decomposes a data matrix containing *n* rows (typically genes) and *m* columns (typically tumor samples) into *p* patterns across samples related to meta-pathways (gene-level amplitude estimates of activity). Meta-pathway activity associated with these patterns is defined as the Z-score of the pattern, estimated as the ratio of the mean pattern to the standard deviation of the pattern estimated with CoGAPS (Methods S2). These meta-pathway activity estimates are then rescaled to have a maximum value of 1 to facilitate visualization of sample associations across the inferred meta-pathways.

We applied the CoGAPS matrix factorization algorithm to simultaneously infer patterns associated with meta-pathway activity from a combined data matrix **D** containing gene expression data for *n_E_* genes and log transformed β values for DNA methylation of *n_M_* genes for the same set of *m* samples, analogous to [10], [11]. Uncertainties of the gene expression data were assumed to be 10% of the signal, and for DNA methylation derived from a normal approximation to the beta distribution [12]. This error model enables CoGAPS to find patterns that decay to zero for those samples, which reflects epigenetic silencing of gene expression.

As in [13], we limited the matrix factorization to genes annotated as transcription factor targets in TRANSFAC [14] identified from ASAP [7]. We also included DNA methylation values for transcription factors because of their expected modification of TF target expression, leaving *n_E_* = 972 and *n_M_* = 892 genes. CoGAPS was run for a burn-in period of 10^8^ iterations (required for all MCMC algorithms) and then statistics were computed over 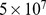 iterations. Results are reported for total number of patterns *p* ranging from two to five based upon inferred pattern robustness and persistence [15], [16] (Methods S2).

### Associating CoGAPS Patterns with Clinical Subtypes

Because CoGAPS does not encode clinical information in the factorization, meta-pathway activity was associated with clinical phenotypes using linear models. P-values associating meta-pathway activity with each phenotype, correcting for multiple-testing, were computed using the R package multcomp [17]. These were compared to hierarchical clustering computed from sample-by-sample Pearson correlation matrices. We cut the resulting tree into *k* clusters, where *k* ranges from two to six. A Fisher exact test was applied to quantify the association of each cluster with the clinical variables (Table S1).

### Gene Set Analyses

We applied gene set analyses to link the inferred metapathways to curated pathways, established as pertinent to human cancers. Epigenetic modulation of gene expression in the meta-pathways was quantified by inverse variance weighting the CoGAPS amplitudes of corresponding genes in the DNA methylation and gene expression amplitude matrices. Enrichment of canonical pathways from MSigDB (C2:CP; i.e., KEGG, Biocarta, and Reactome) [18] in the epigenetically modulated meta-pathways was computed using permutation tests [9], [19].

### Validation Cohort and RT-PCR Assays

RNA from the independent cohort of 32 HNSCC and 15 UPPP was transcribed to cDNA with the High Capacity cDNA Reverse Transcription Kit from Applied Biosystems (Carlsbad, CA), according to manufacturer’s instructions. Subsequently, 15 ng of template was used and quantitative RT-PCR was performed to confirm *GLI1* expression in these samples. Samples were run in triplicate in 96-well plates using the Step-One real-time PCR machine from Applied Biosystems. Taqman assays were used for both *GLI1* and β-Actin (Hs01110773 and Hs01060665_g1, respectively), as per manufacturers instructions (Applied Biosystems, CA). Two µl of the cDNA was used per replicate. Relative change in expression between normal and tumor were computed with one-sided t-statistics on ΔCt values.

We compared *GLI1* and β-catenin (*CTNNB1*) expression from level 3 RNA-seq v2 data in the Cancer Genome Atlas (TCGA) for the 28 normal, 29 HPV-positive, and 179 HPV-negative HNSCC samples available as of August, 2012. We applied univariate t-tests on read counts from Illumina HiSeq 2000 RNA Sequencing that were RSEM normalized [20] and log transformed.

## Results

### Inferred Meta-pathways Distinguished HNSCC Clinical Phenotypes

**Figure 1:**
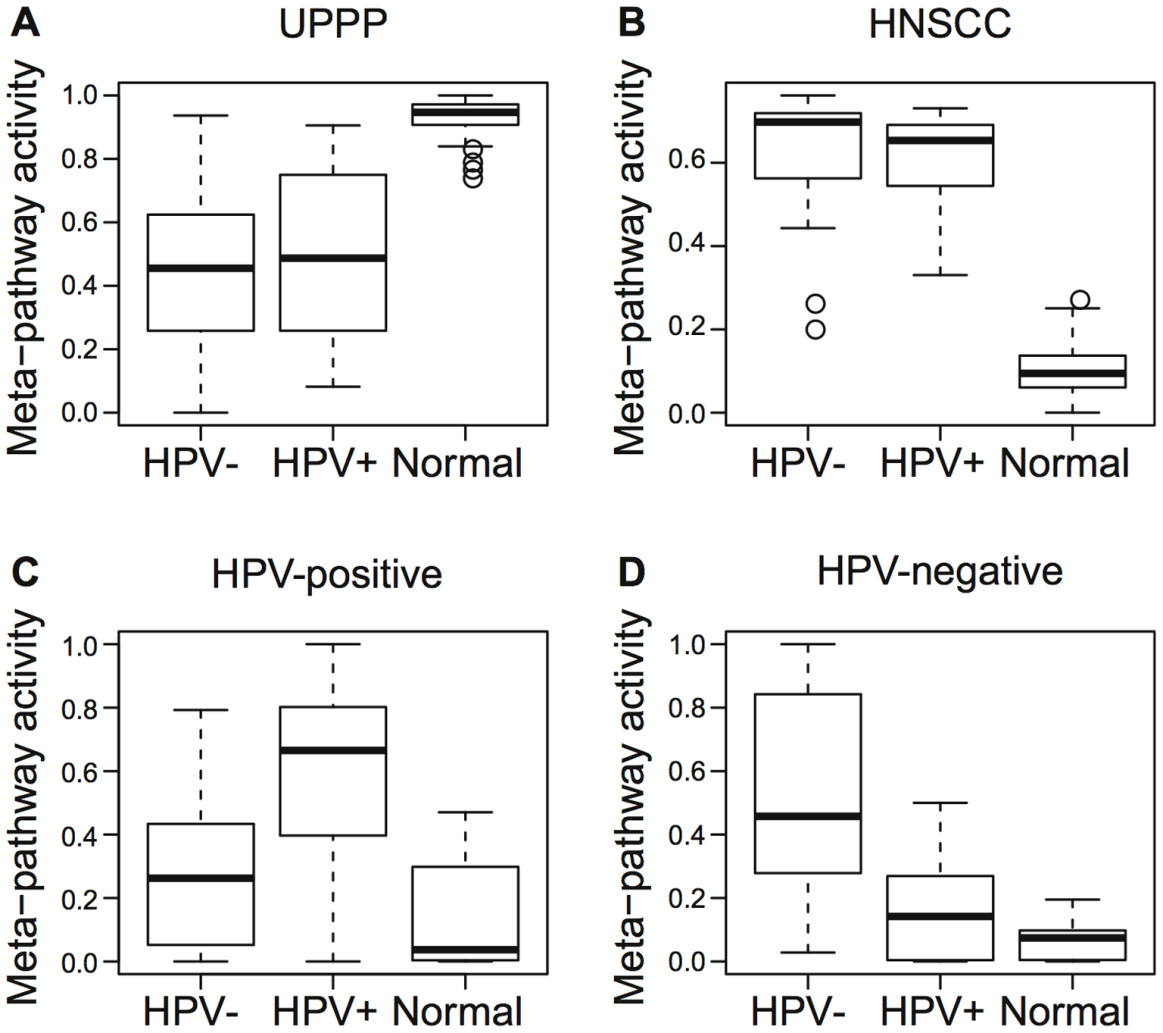
Meta-pathway activity identified in 44 HNSCC and 25 UPPP samples. Relative activity of meta-pathways associated with (a) UPPP, (b) HNSCC, (c) HPV-positive, and (d) HPV-negative samples.

We applied CoGAPS to infer combinations of genes (meta-pathways) with coordinated DNA methylation and gene expression changes across subsets of 44 HNSCC samples and 25 normal samples from uvulopalatopharyngoplasty (UPPP) (clinical attributes in Table 1). CoGAPS assigns each sample a magnitude for “meta-pathway activity” that indicates the similarity of that sample’s DNA methylation and gene expression changes other subsets of samples with similar genomic signatures (Methods S2). These subsets will be analyzed as disease subtypes. Even though CoGAPS does not encode clinical information about the samples, the analysis isolated meta-pathway activity that significantly distinguished normal (Figure 1(a); p = 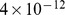) and tumor samples (Figure 1(b); p<10^−16^). The samples from smokers tend to have similar DNA methylation and gene expression changes to those in the meta-pathway associated with tumor samples (p = 0.001; Figures S1).

The meta-pathway analysis further distinguished meta-pathways associated with HPV status, clearly distinguishing HPV-positive (Figure 1(c); p = 10^−6^) and HPV-negative samples (Figure 1(d); p = 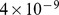). Activity in the HPV-positive meta-pathway is significantly higher than those for female samples (p = 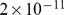) (Figure S1). We attribute this trend to similarities arising from the predominance of male samples (11 male versus 2 female) in the HPV-positive training cohort. Moreover, the association with HPV status remains statistically significant in a multivariate model accounting for both HPV status and gender (p = 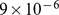), resulting from a larger pattern value for HPV-positive male samples than male samples that are either HPV-negative or normal. Each of these meta-pathways was identified from similar analyses on DNA methylation data alone, but only the HPV-negative meta-pathway was identified in gene expression data alone (Figure S2).

### Meta-pathway Analysis More Strongly Distinguishes Clinical Phenotypes than Hierarchical Clustering

**Figure 2:**
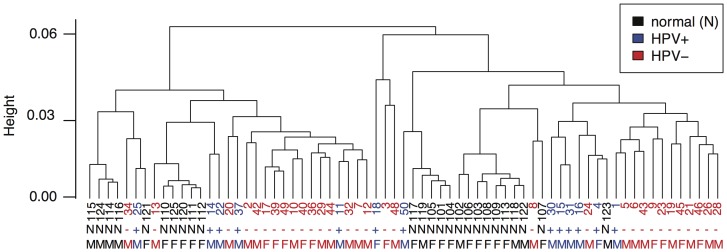
Clustering combined DNA methylation and gene expression data from 44 HNSCC samples and 25 UPPP samples. Patterns identified with hierarchical clustering for sample-by-sample Pearson correlations for combined gene expression and log transformed DNA methylation data, colored by whether samples are normal (black, labeled “N”), HPV-positive (blue, labeled “+”) and HPV-negative (red, labeled “−”) samples.

For comparison, we clustered the samples using the gene expression and DNA methylation data sets (Figure 2; Table S1), each of which has been subset to the same genes that were used for the meta-pathway analysis (Methods Section). Hierarchical clustering on the combined gene expression and DNA methylation dataset also significantly separated tumor and normal samples (p-value of 0.02). Unlike the meta-pathway analysis, the clustering did not significantly distinguish HPV status or tumor site from the combined DNA methylation and gene expression dataset, nor was the split observed in the dendrogram significantly related to gender differences (Figure 2; Table S1). However, clustering performed on expression data alone (Figure S3) did significantly separate HPV-positive and HPV-negative samples (p-value of 0.05) suggesting the phenotypic differences between HPV+ and HPV− may be predominantly driven by the transcriptional deregulation rather than epigenetic changes. On the other hand, clustering of the DNA methylation data alone associated with differences in smoking status (p-value of 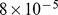), race (p-value of 0.01), gender (p-value of 0.02), and tumor site (p-value of 0.02), but not HPV-status.

### Enriched Pathways Reflect Known HNSCC Biology and Identify Novel Activity of the Hedgehog and Wnt Pathways Specific to HPV-negative HNSCC

Enrichment analysis was applied to identify epigenetically driven pathway modulations in each of the meta-pathways identified in the HNSCC tumors (Table 2). This analysis identified enrichment of gene sets generally attributed to cancers in patterns associated with HNSCC, including KEGG cancer pathways, ERBB, and MAPK/EGFR signaling pathway activity. The analysis also implicated Hedgehog signaling in HNSCC.

**Table 2:** Pathway enrichment Summary of the pathways that were significantly enriched (p<0.05) in each meta-pathway associated with HNSCC.

PathwayDatabase	Tumor Meta-pathway	HPV-Positive Meta-pathway	HPV-Negative Meta-pathway
**KEGG**	ERBB signaling pathway, Hedgehog signalingpathway, Adherens junction, Epithelial cellsignaling in *H. pylori* infection, Pathogenic*E. coli* infection, Endometrial cancer,Basal cell carcinoma	Glycolysis and gluconeogensis, Arginine and proline metabolism, Glutathione metabolism, ERBB signaling pathway, GNRH signaling pathway, Focal adhesion, Adherens junction, Prion diseases, Leishmania infection, Endometrial cancer	ERBB signaling pathway, WNT signaling pathway, Hedgehog signaling pathway, Axon guidance, Adherens junction, Pathogenic *E. coli* infection, Pathways in cancer, Endometrial cancer, Prostate cancer, Thyroid cancer, Basal cell carcinoma
**Biocarta** **Pathways**	P35 Alzheimers, Gleevec, Keratinocyte, PYK2,MAPK, NGF, Cardiac EGF	Biopeptides, EGF, EPO, ERK, P53 Hypoxia,IGF1, GSK3, Insulin, NGF, GPCR, Toll	AT1R, CDMAC, P35 Alzheimers, RACCYCD, Gleevec, Integrin, Keratinocyte, PYK2, MAPK, ETS, Cardiac EGF, WNT
**Reactome**	NCAM signaling for neurite out growth, SLCmediated transmembrane transport,transmembrane transport of small molecules	Basigin interactions, Diabetes pathways,Gluconeogenesis, Glucose metabolism,Metabolism of carbohydrates, NF KB isactivated and signals survival, P75 NTRreceptor mediated signaling, P75NTR signalsvia NFKB, Regulation of Insulin like growthfactor activity by insulin like growth factorbinding proteins, Toll receptor cascades	Axon guidance, Clathrin derived vesicle budding, Membrane trafficking, Amino acids, Toll like receptor 3 cascade

Similar pathway enrichment was inferred for HPV-negative tumors. Notably the Hedgehog enrichment was unique to this class of tumors. Moreover, Wnt pathway enrichment was also uniquely identified in HPV-negative tumors, which overlaps considerably with the Hedgehog pathway but had not previously been associated with HNSCC.

Several of the pathways inferred in HNSCC tumors are significantly enriched in HPV-positive tumors, including ERBB and MAPK/ERK signaling. Unique to HPV-positive tumors are pathways related to immune response (Prion diseases and Leishmania infection) and pathways related to metabolic processes. Moreover, further pathway analyses of gene expression data in the HPV-positive meta-pathway identified enriched cell cycle activity in HPV-positive tumors (Table S2).

### GLI1 Overexpression Confirms Hedgehog Pathway Activity in HNSCC Tumors

Although the analysis implicated statistically significant epigenetic modulation of the Wnt and Hedgehog pathways in HPV-negative HNSCC, discerning specific pathway activity in these samples was complicated by the significant overlap in the gene sets annotated to these pathways. Therefore, we further analyzed gene expression of β-catenin (*CTNNB1*) as a standard marker of Wnt activity [21] and *GLI1* as a standard marker of Hedgehog activity [22]. We analyzed probe-level expression values to account for alternative transcription of *GLI1*, previously reported for Hedgehog pathway activity in basal cell carcinoma [23]. Whereas β-catenin was significantly overexpressed in both HPV-positive samples and HPV-negative samples relative to normal (minimum one-sided p-values of 0.009 and 0.01, respectively), fold changes were modest (maximum of 0.7 over normal samples). On the other hand, *GLI1* was only significantly overexpressed in HPV-negative samples (minimum one-sided p-values of 0.04 in HPV-negative samples and of 0.07 in HPV-positive samples) compared to the normal tissue. Moreover, several samples had *GLI1* expression significantly above the maximum expression in normal samples (Figure S4; maximum fold change above normal range of 2 in HNSCC tumors). The *GLI1* overexpression of at least 0.5 log fold change above the normal occurred in six of the HPV-negative tumors, but only one of the HPV-positive tumors. The difference in *GLI1* expression between HPV-positive and -negative failed to reach the statistical significance. Nonetheless, it is notable that the only HPV-positive sample with *GLI1* overexpressed above levels observed for normal samples was from a smoker with cancer in the larynx, not typical of the clinical characteristics of HPV-positive HNSCC (Table 1).

### RT-PCR Validation of Hedgehog Pathway Activation in HNSCC

**Figure 3:**
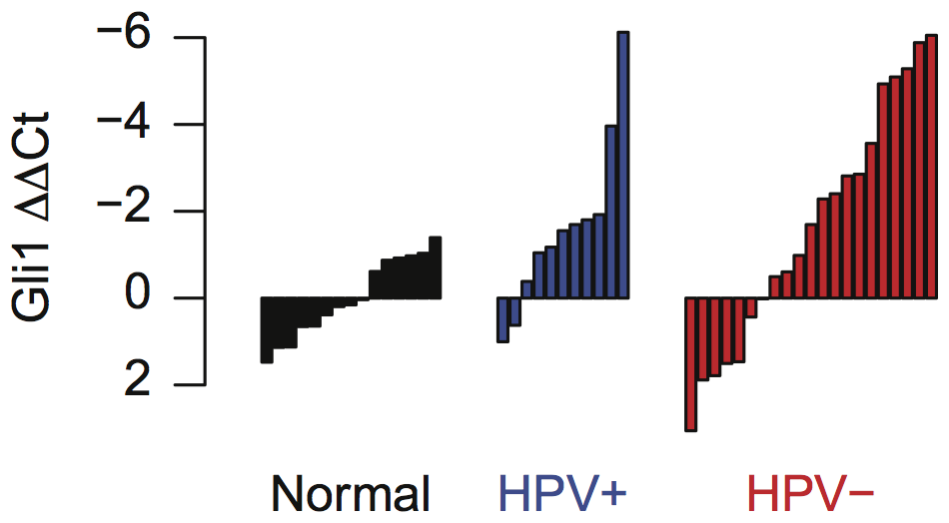
GLI1 expression in the validation cohort. (a) GLI1 expression in validation cohort measured with RT-PCR. 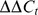 values are computed relative to the mean 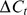 count of for normal samples.

We used QRT-PCR to measure *GLI1* expression in a small, independent cohort of 32 HNSCC (11 HPV-positive and 21 HPV-negative) and 15 UPPP samples as a readout to validate Hedgehog pathway activation in these samples [22]. This analysis confirmed that *GLI1* was significantly overexpressed in all HNSCC samples compared to normal mucosa from non-cancer affected individuals, with a mean fold changes of 3.1 above normals (one-sided p-value of 0.001 from a t-test; Figure 3). We noted a trend for a subpopulation of *GLI1* overexpression in HNSCC that did not reach statistical significance.

### TCGA Data Confirms Specificity of GLI1 Overexpression in HPV-negative HNSCC

**Figure 4:**
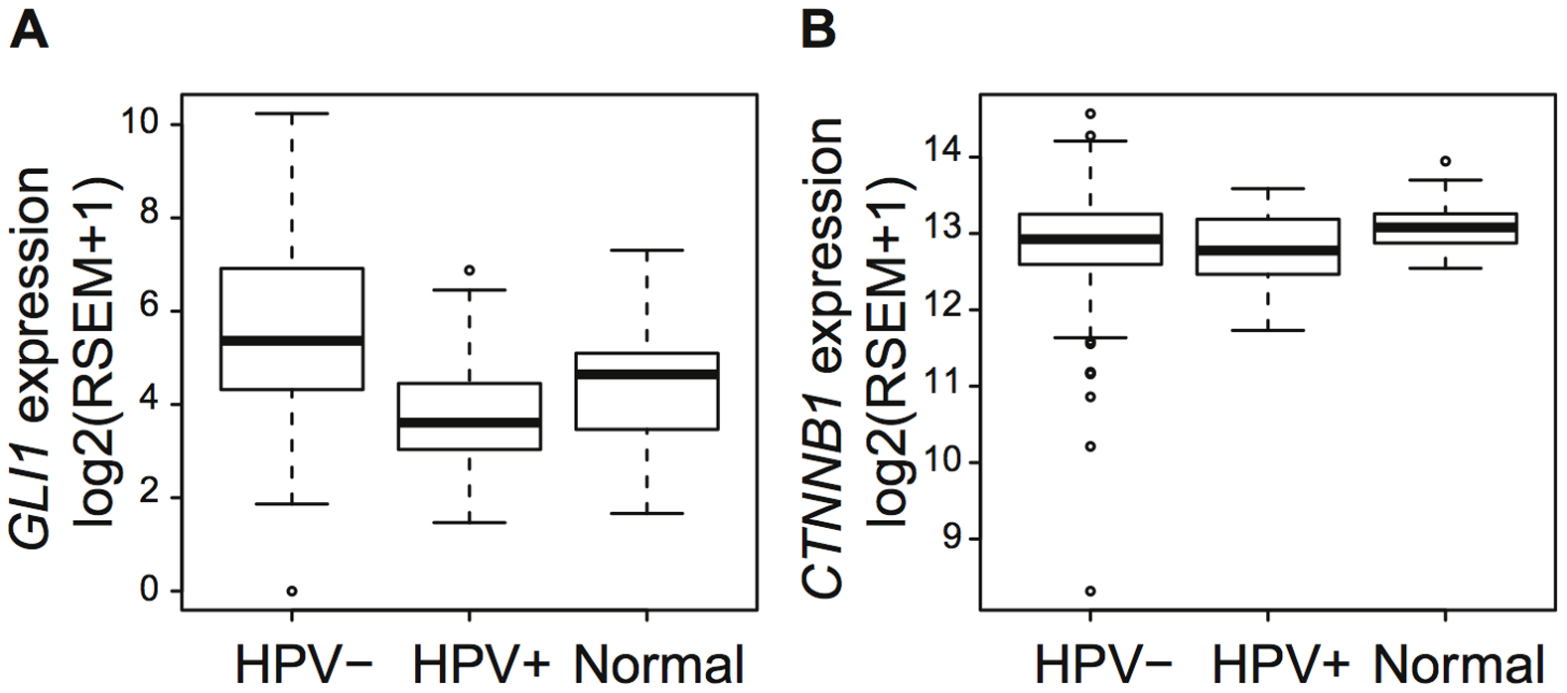
GLI1 and CTNNB1 expression in TCGA. Boxplots of (a) GLI1 and (b) CTNNB1 expression in TCGA RNA-seq data.

To further explore the relationship of HPV status to Hedgehog activation, we employed RNA-seq data on a larger cohort from TCGA (The Cancer Genome Atlas; multi-institutional, curated samples with HPV status confirmed with next generation sequencing). TCGA data confirmed that *GLI1* was significantly overexpressed in the 244 HPV-negative samples relative to the 35 HPV-positive (p-value of 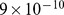, Figure 4(a)), suggesting our 32-sample cohort may have been too small to detect the difference between HPV-positive and -negative samples using only one gene. Moreover, 18% of genes in the KEGG Hedgehog pathway are significantly overexpressed and 11% significantly hypomethylated, including *GLI1*, in TCGA (Table S3). Consistent with [24], the Wnt pathway marker *CTNNB1* is significantly underexpressed in the 279 HNSCC as compared to the 37 matched normal samples (p-value of 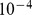) in TCGA. Differences between HPV-positive and HPV-negative samples do not reach statistical significance (p-value of 0.4) (Figure 4(b)). We note the lack of differential expression of *CTNNB1* in TCGA is in contrast to findings in our discovery cohort. Such differences may arise from technological differences in gene expression measurements between RNA-seq and arrays or site-specific expression changes, arising from comparison to matched normal samples in TCGA and to unmatched UPPP samples in the discovery cohort. Expression of *GLI1* and *CTNNB1* do not associate with gender, in spite of the substantial gender imbalance in HPV-positive HNSCC samples from TCGA (Figure S5).

## Discussion

Although not encoded in the analysis, meta-pathways identified from integrated DNA methylation and gene expression distinguished normal samples, HNSCC tumor samples, gender, HPV status, and tumor site, which were not distinguished robustly with hierarchical clustering. This improvement is similar to that observed with other non-negative matrix factorization (NMF) algorithms over clustering for subtype identification [25]. We note that this represents the first application of Bayesian non-negative matrix factorization algorithms including Bayesian Decomposition [26] or CoGAPS [9] to such subtype identification. We anticipate that these Bayesian techniques yield similar improvements in the biological relevance of meta-pathways associated with these subtypes that we have previously documented in pattern identification [27], [28], consistent with the improvements over hierarchical clustering observed in this study.

The subtypes identified on the integrated CoGAPS analysis were not identified in similar analyses on gene expression data alone. Moreover, this analysis enhanced the anti-correlation between gene expression and DNA methylation. Therefore, changes in DNA methylation drive the meta-pattern analysis while inducing transcriptional changes that distinguish clinical subtypes of HNSCC. We anticipate that these improvements arise by explicitly encoding epigenetic silencing of gene expression by use of the log transform. Nonetheless, future studies should compare the effect of alternative mappings of DNA methylation such as the logistic transform [12], on the inference of epigenetic silencing and subtype identification.

We note that the inferred meta-pathways may be confounded by differences in subsites of each of these HNSCC subtypes because of the tissue-specific nature of DNA methylation [29]. The imbalance in subtypes of HNSCC may further confound inferred differences, including the notable overrepresentation of Caucasian males in HPV-positive HNSCC reflected in our sample cohort and in TCGA samples and also associated with the HPV-specific meta-pathways. Both limitations would be best addressed by future prospective studies that employ comprehensive genomic profiling of balanced patient populations. These studies should also incorporate normal tissues from diverse sites throughout the head and neck region because the substantial field cancerization effect in HNSCC [30] limits the utility of matched normal samples in TCGA.

In spite of the limitations in study population, the association of the meta-pathways with pathways that have established links to HNSCC supported validity of the inferred meta-pathways. For example, the algorithm identified enrichment signaling downstream of EGFR ubiquitously in HNSCC, consistent with pervasive overexpression of EGFR previously documented in HNSCC [31]. Moreover, EGFR overexperession has been associated with poor prognosis in HNSCC [32] and is the target of the antibody cetuximab, which has been FDA approved for the management of HNSCC.

The meta-pathway for HPV-positive tumors is also associated with immune responses to viral infection and upregulation of cell cycle events, which has been observed along with p16 (CDKN2A) upregulation in HPV-positive tumors due to Rb deregulation caused by HPV E7 oncoprotein [33], [34]. Moreover, the upregulation of metabolic processes including notably pathways associated with the arginine and proline metabolism is consistent with recent findings that HPV-positive HNSCC has a high expression level of argininosuccinate synthetase suggesting that arginine metabolism is important in HPV+ tumors [34]. This analysis notably associated Hedgehog activity specific to HPV-negative HNSCC, confirmed as associated to HPV-status rather than gender in independent samples from TCGA. Exon-specific differences observed for *GLI1* in the discovery cohort were consistent with previously reported alternative transcription of this gene in basal cell carcinoma [23] and the lack of detection in previous studies performed on previous HNSCC array studies [35]–[39].

The meta-pathway CoGAPS analysis also inferred pathways in subsets of patients previously not described in HNSCC, including pathways associated with subsets of HPV-negative tumors. HPV-negative tumors carry a significantly worse prognosis than HPV-positive tumors [2], and therapy for advanced HNSCC is currently at the limits of toxicity. Recent clinical trials have been developed to provide means of de-escalation for HPV-positive tumors. However, there have been a paucity of effective agents for HPV negative tumors, and developing clinical trials have recently focused on the addition of surgical intensification of therapy in combination with cytotoxic chemotherapy and radiation. Notably, the analysis identified coordinated methylation and expression changes in the Hedgehog signaling pathway in HPV-negative tumors, which we confirmed by differential expression of the Hedgehog target *GLI1* [22]. The nature of *GLI1* overexpression was consistent with patterns identified with outlier based statistics previously used to identify sample-specific oncogenes in HNSCC [40] and pathway level changes [41]. Promisingly, RT-PCR confirmed the increase of *GLI1* expression levels in HNSCC tumors over normals and RNA-seq from TCGA confirmed higher *GLI1* expression in HPV-negative samples. The increased expression of *GLI1* in HNSCC tumors in this study is consistent with observed *GLI1* overexpression in HNSCC [24], [42], [43] and our previously published data showing that high expression of nuclear *GLI1* is associated with poor survival and distant metastasis [24].

*GLI1* is a transcription factor and a downstream target of the canonical Hedgehog signaling pathway. After hedgehog ligand binding, the transmembrane receptor Patched de-represses Smoothened, which in turn activates transcription of target genes such as *GLI1*. However, *GLI1* can also be activated by Smoothened-independent, non-canonical mechanisms [44], [45]. Recently, the first-in-class Smoothened antagonist, vismodagib (GDC0449), gained FDA approval for the treatment of basal cell carcinoma. Basal cell carcinomas are largely characterized by mutations in the Hedgehog signaling axis that render the pathway constitutively active, resulting in remarkable single-agent efficacy demonstrated with vismodagib [46]. Robust single-agent efficacies have also been seen in medulloblastomas, where nearly one-third of cases are associated with constitutive activation of the Gli1 transcription factor through similar oncogenic mutations in the Hedgehog pathway [47]. The mechanism and role of Gli1 activation in HNSCC is probably distinct from these two examples because no such mutations have been identified [48], [49]. Such identification of pathway activation in specific subsets of HPV-negative HNSCC patients may allow for selection of specific targeted agents and aid in clinical trial design. However, further mechanistic studies are required to delineate canonical and non-canonical activation of Hedgehog reflected by *GLI1* expression in HPV-negative HNSCC prior to implementing such future clinical trials.

## Supporting Information

Figure S1 **Meta-pathway activity identified in 44 HNSCC and 25 UPPP samples.** Relative activity of meta-pathways associated with (a) UPPP, (b) HNSCC, (c) HPV-positive, and (d) HPV-negative samples. Symbols represent subsite of each sample, shading smoking status, and color gender according to the figure legend. The p-values on each figure represent one-sided, multivariate p-values comparing differences in the indicated groups.
(PDF)

Figure S2 **Dependence of meta-pathways on DNA methylation or gene expression.** Correlation of the meta-pathway activity for each of the patterns linked to UPPP (Figure 1(a)), HNSCC (Figure 1(b)), HPV-positive (Figure 1(c)), and HPV-negative (Figure 1(d)) samples to patterns found in DNA methylation data alone (blue) or gene expression data alone (yellow).
(PDF)

Figure S3 **Clustering DNA methylation or gene expression data.** Hierarchical clustering on sample-by-sample Pearson correlations computed for (a) gene expression data and (b) log transformed DNA methylation data. Samples are colored black if normal, blue if HPV-positive, and red if HPV-negative.
(PDF)

Figure S4 **GLI1 and CTNNB1 expression in discovery cohort.** GLI1 expression in (a) HPV-negative samples and (b) HPV-positive samples across GLI1 probes relative to normals (expression bounded by black lines) for each core probe measured with the HuEx array. (c) Genomic location of GLI1 exons measured and (d) waterfall plots of average GLI1 expression in each exon relative to the mean expression values for normal samples. (e)-(h) are as for (a)-(d) for CTNNB1.
(PDF)

Figure S5 **GLI1 and CTNNB1 expression in TCGA by gender.** Scatter plot of expression values for (a) GLI1 and (b) CTNNB1 from TCGA RNA-sequencing data, divided by gender, tumor and HPV-status of samples.
(PDF)

Table S1 **Clinical attributes of clusters.** Table associating clinical variables with clusters identified from DNA methylation and/or gene expression data.
(XLSX)

Table S2 **Meta-pathway set enrichment for gene expression data.** Table containing gene set statistics computing from the meta-pathway values for gene expression data.
(CSV)

Table S3 **Differential expression and methylation of KEGG Hedghog pathway members in** **TCGA.**
(XLSX)

Methods S1 **R code.** ZIP archive containing R code used to for the analysis in this manuscript. The README file in the archive describes each of the files contained therein. To fully reproduce the results, the scripts should be run in the following order (1) Preprocessing.R, (2) RunMEGAPSReplicateThreshold.R, and (3) Postprocessing.R. Note that the CoGAPS analysis and associated gene set statistics are stochastic algorithms, so results may differ slightly from values reported here though qualitative results will remain unchanged.
(ZIP)

Methods S2 **Supplemental Methods.**
(PDF)
