# Supplementary figures and images for: Preferential Activation of the Hedgehog Pathway by Epigenetic Modulations in HPV Negative HNSCC Identified with Meta-Pathway Analysis
Source: PLoS One. 2013 Nov 4;8(11):e78127. doi: 10.1371/journal.pone.0078127 (PMC3817178; doi:10.1371/journal.pone.0078127)

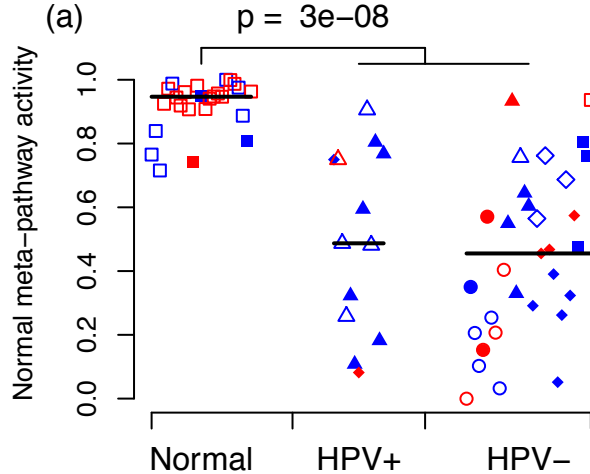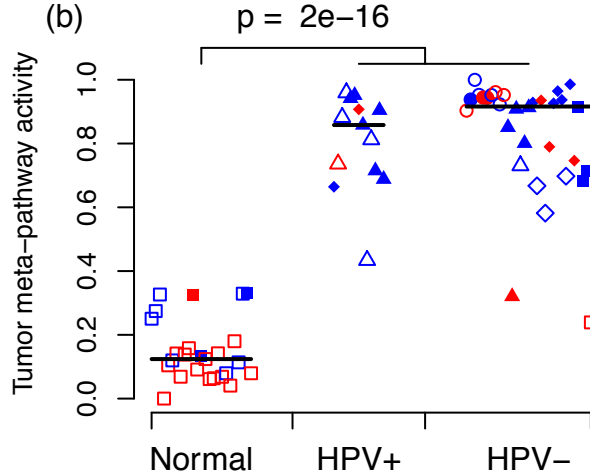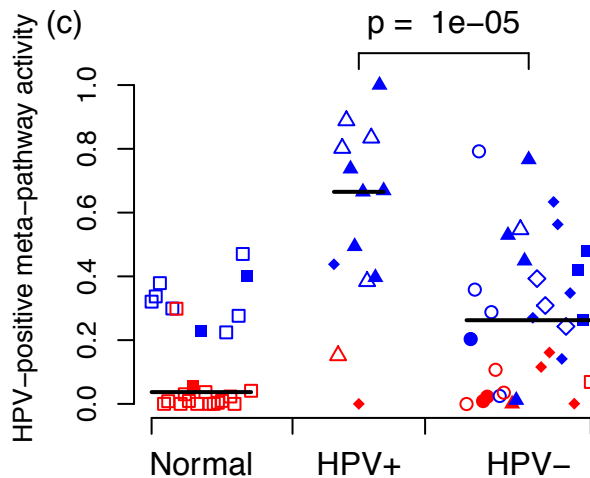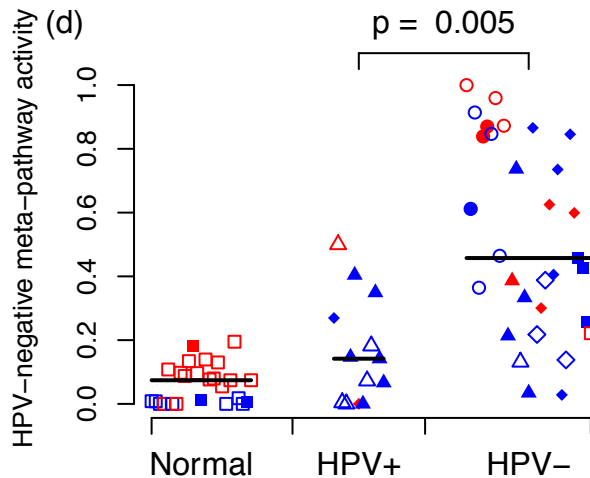

○ Oral Cavity  
△ Oropharynx  
◇ Larynx  
□ Hypopharynx / UPPP

■ Smoking  
□ Non-smoking

— Male  
— Female

Supplement: Figure S1 — Meta-pathway activity identified in 44 HNSCC and 25 UPPP samples. Relative activity of meta-pathways associated with (a) UPPP, (b) HNSCC, (c) HPV-positive, and (d) HPV-negative samples. Symbols represent subsite of each sample, shading smoking status, and color gender according to the figure legend. The p-values on each figure represent one-sided, multivariate p-values comparing differences in the indicated groups. (PDF) [file pone.0078127.s001.pdf]

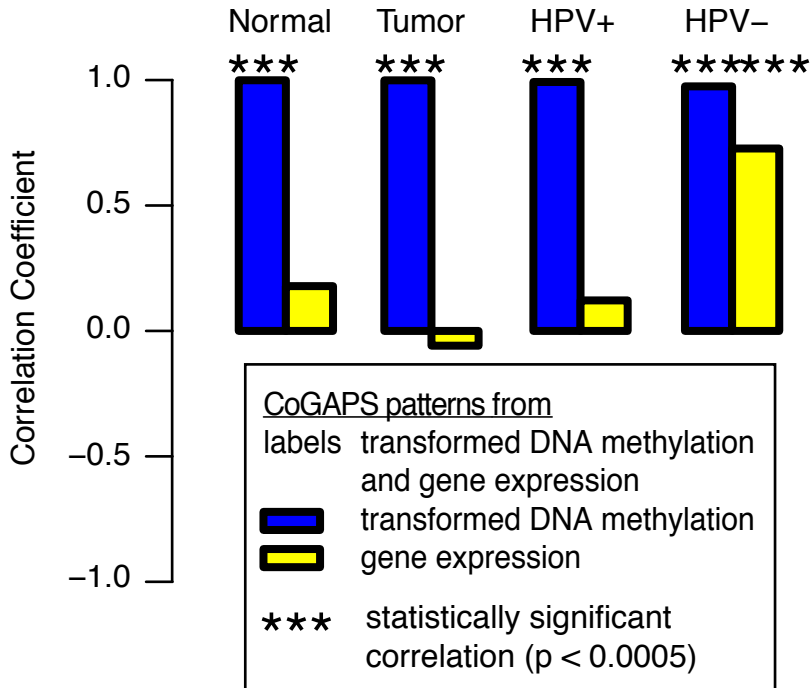

Supplement: Figure S2 — Dependence of meta-pathways on DNA methylation or gene expression. Correlation of the meta-pathway activity for each of the patterns linked to UPPP (Figure 1(a)), HNSCC (Figure 1(b)), HPV-positive (Figure 1(c)), and HPV-negative (Figure 1(d)) samples to patterns found in DNA methylation data alone (blue) or gene expression data alone (yellow). (PDF) [file pone.0078127.s002.pdf]

A

GLI1. RNA seq data

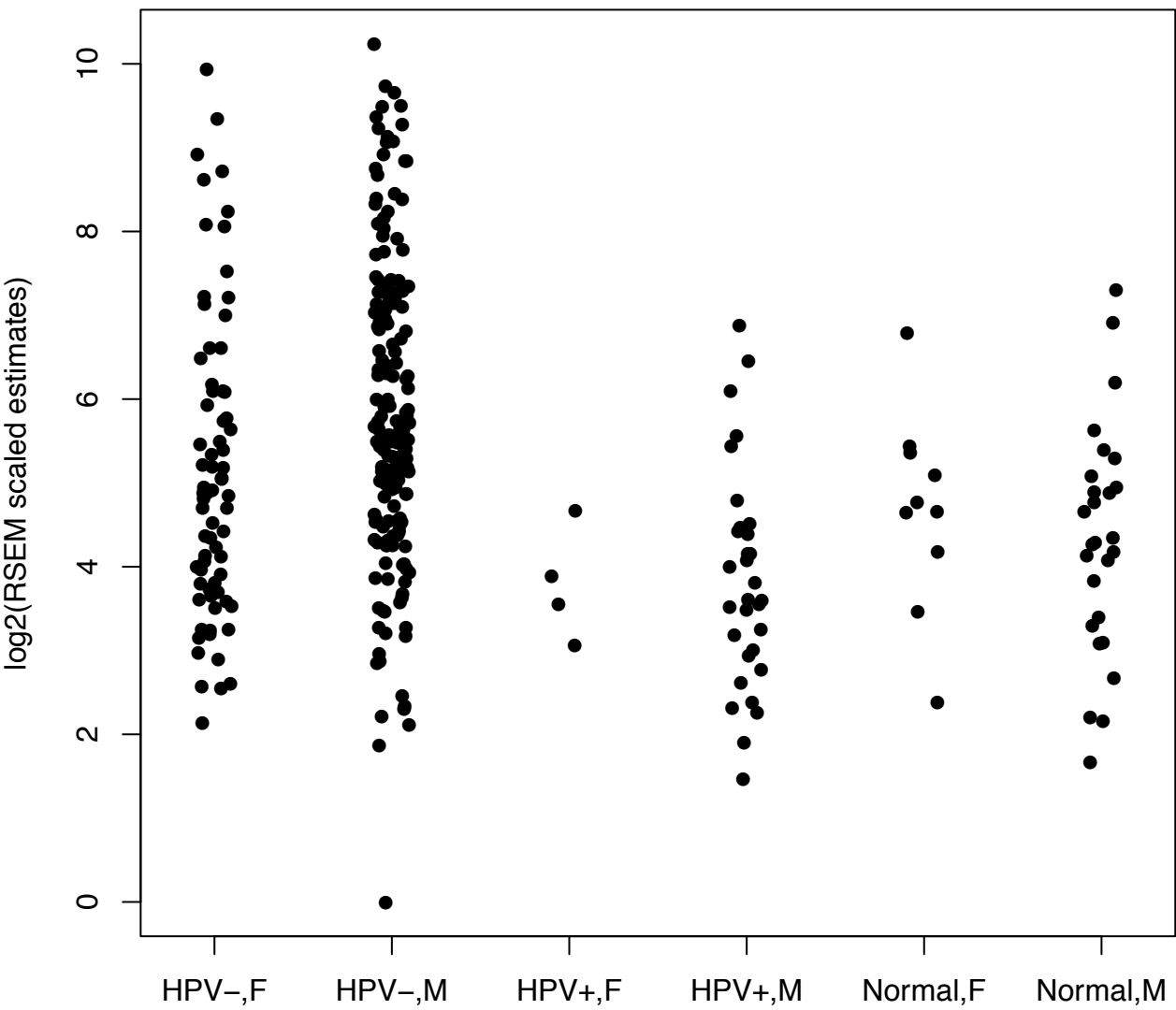

B

CTNNB1. RNA seq data

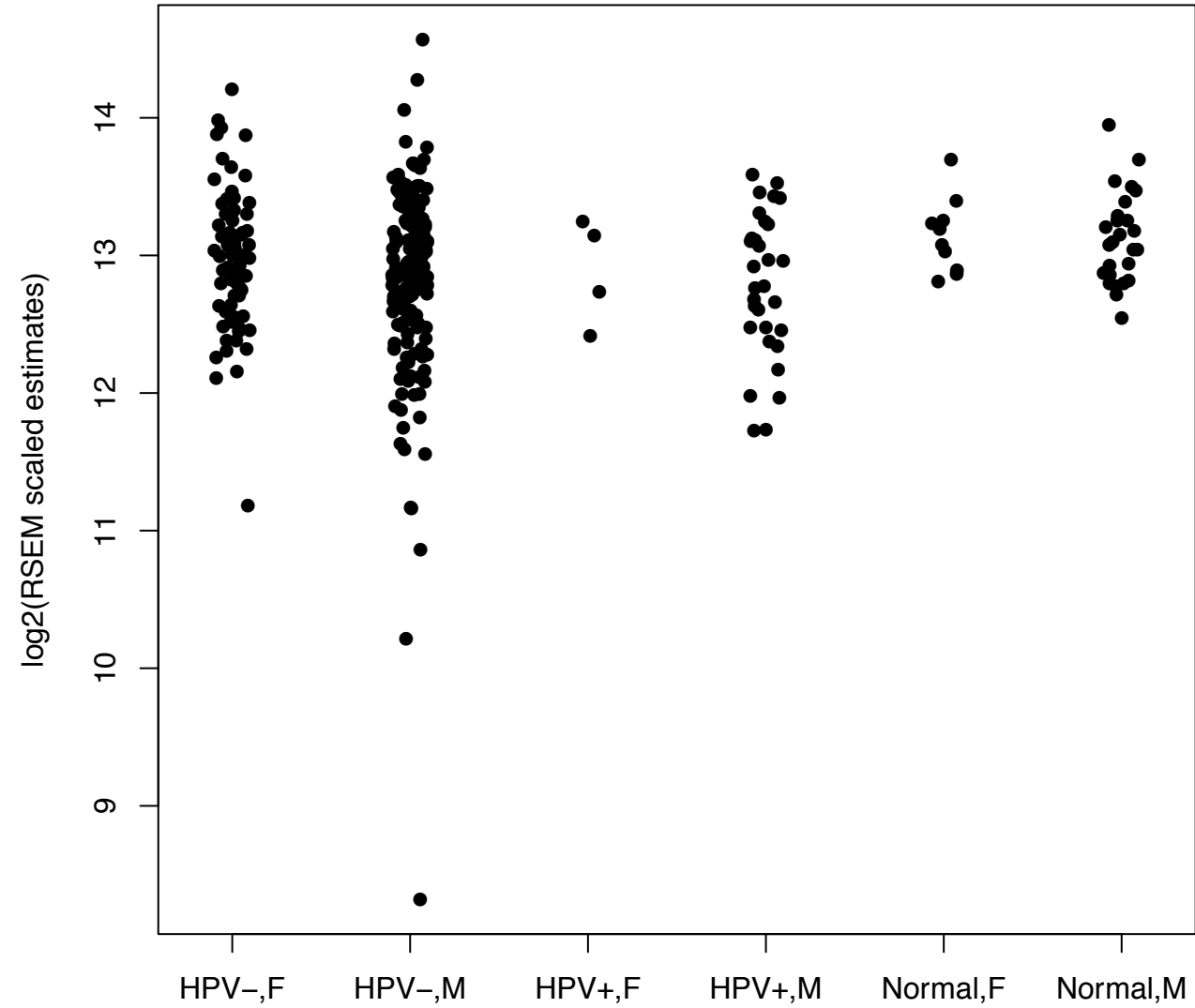

Supplement: Figure S5 — GLI1 and CTNNB1 expression in TCGA by gender. Scatter plot of expression values for (a) GLI1 and (b) CTNNB1 from TCGA RNA-sequencing data, divided by gender, tumor and HPV-status of samples. (PDF) [file pone.0078127.s005.pdf]
