# Supplementary material for: Preferential Activation of the Hedgehog Pathway by Epigenetic Modulations in HPV Negative HNSCC Identified with Meta-Pathway Analysis
Source: PLoS One. 2013 Nov 4;8(11):e78127. doi: 10.1371/journal.pone.0078127 (PMC3817178; doi:10.1371/journal.pone.0078127)
Supplement: Figure S4 — GLI1 and CTNNB1 expression in discovery cohort. GLI1 expression in (a) HPV-negative samples and (b) HPV-positive samples across GLI1 probes relative to normals (expression bounded by black lines) for each core probe measured with the HuEx array. (c) Genomic location of GLI1 exons measured and (d) waterfall plots of average GLI1 expression in each exon relative to the mean expression values for normal samples. (e)-(h) are as for (a)-(d) for CTNNB1. (PDF) [file pone.0078127.s004.pdf]

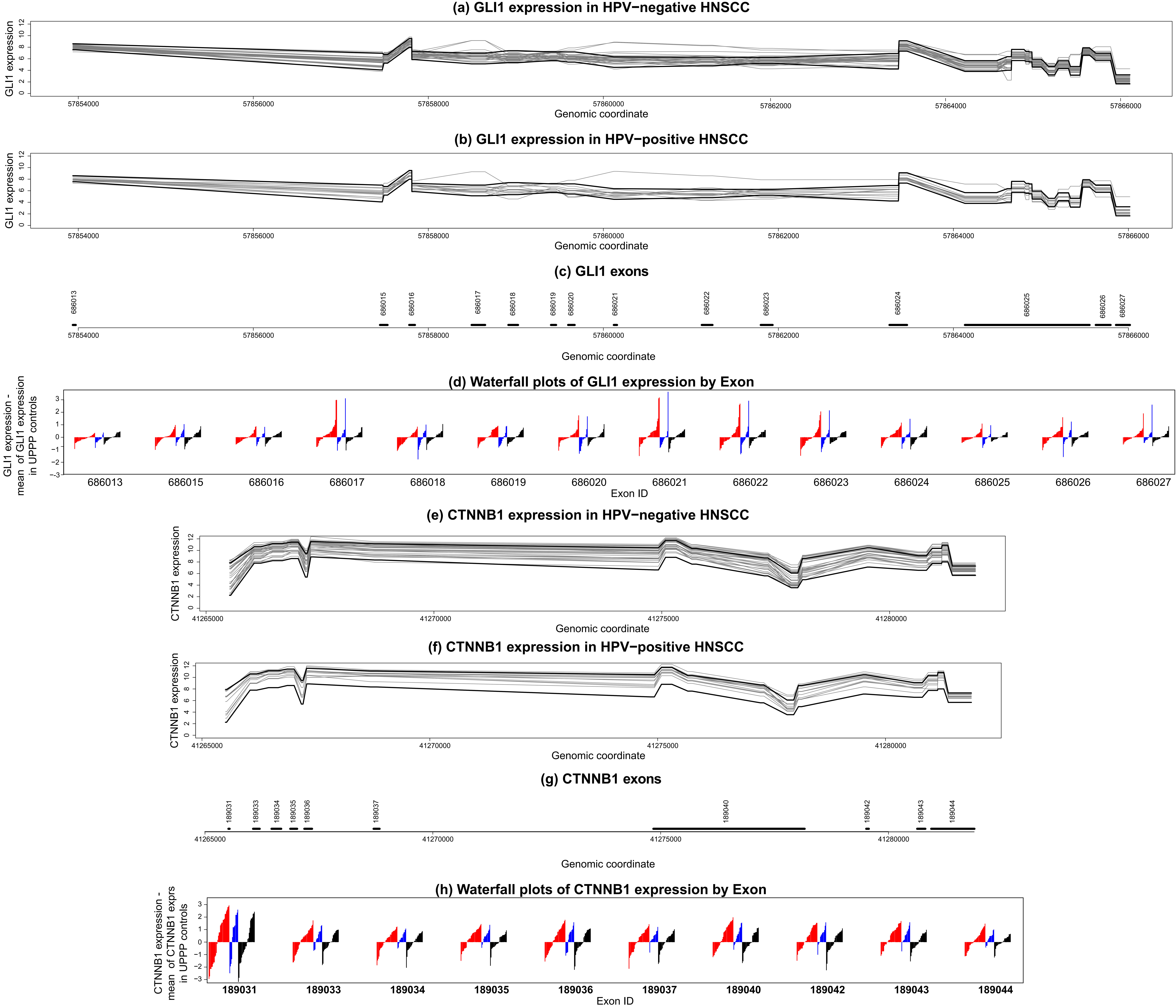
(a) GLI1 expression in HPV−negative HNSCC
GLI1 expression
Genomic coordinate
(b) GLI1 expression in HPV−positive HNSCC
GLI1 expression
Genomic coordinate
(c) GLI1 exons
686013
686015
686016
686017
686018
686019
686020
686021
686022
686023
686024
686025
686026
686027
Genomic coordinate
(d) Waterfall plots of GLI1 expression by Exon
GLI1 expression - mean of GLI1 expression in UPPP controls
Exon ID
(e) CTNNB1 expression in HPV−negative HNSCC
CTNNB1 expression
Genomic coordinate
(f) CTNNB1 expression in HPV−positive HNSCC
CTNNB1 expression
Genomic coordinate
(g) CTNNB1 exons
189031
189033
189034
189035
189036
189037
189040
189042
189043
189044
Genomic coordinate
(h) Waterfall plots of CTNNB1 expression by Exon
CTNNB1 expression - mean of CTNNB1 exprs in UPPP controls
Exon ID
