# Supplementary material for: Preferential Activation of the Hedgehog Pathway by Epigenetic Modulations in HPV Negative HNSCC Identified with Meta-Pathway Analysis
Source: PLoS One. 2013 Nov 4;8(11):e78127. doi: 10.1371/journal.pone.0078127 (PMC3817178; doi:10.1371/journal.pone.0078127)
Supplement: Figure S3 — Clustering DNA methylation or gene expression data. Hierarchical clustering on sample-by-sample Pearson correlations computed for (a) gene expression data and (b) log transformed DNA methylation data. Samples are colored black if normal, blue if HPV-positive, and red if HPV-negative. (PDF) [file pone.0078127.s003.pdf]

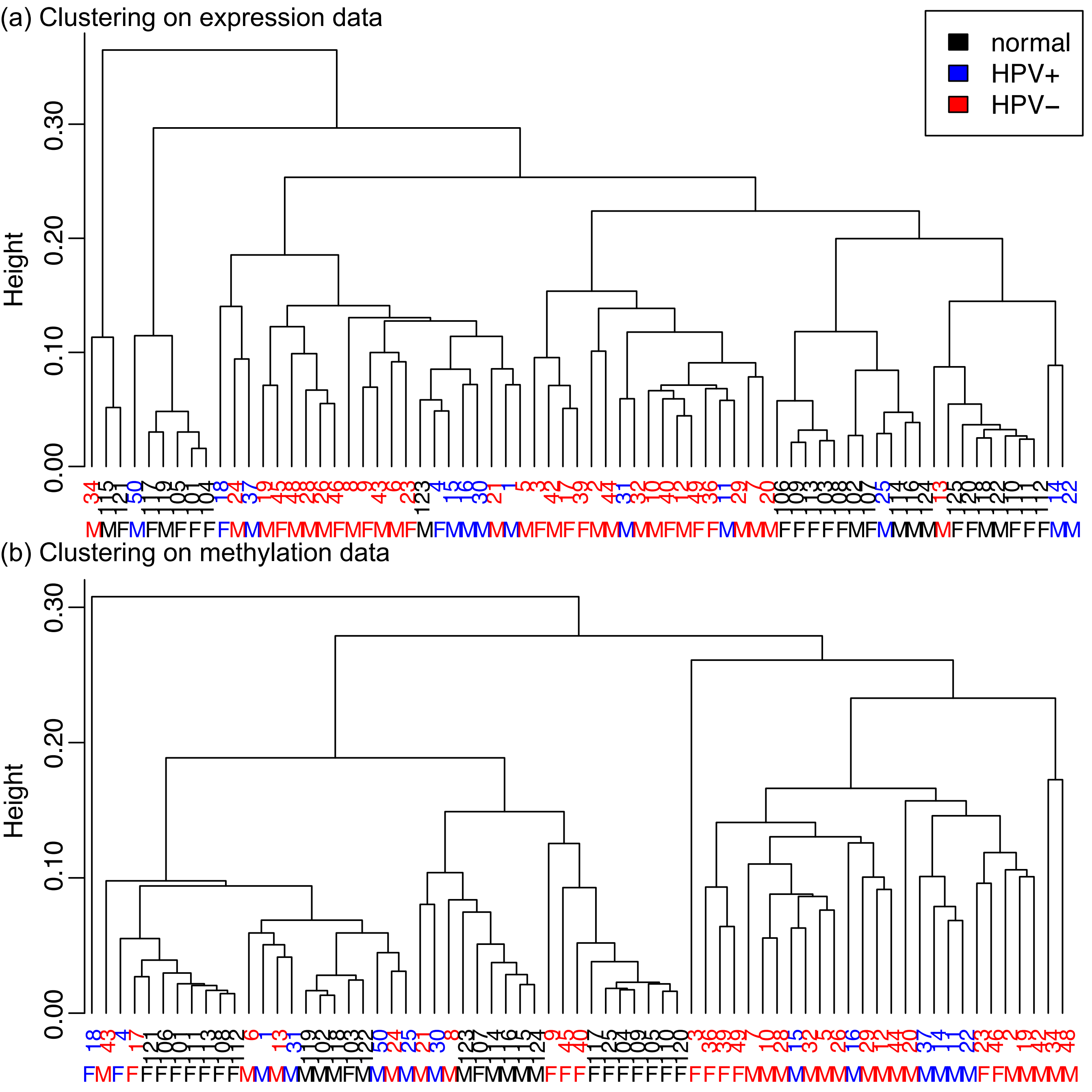

(a) Clustering on expression data
normal
HPV+
HPV−
Height
0.00
0.10
0.20
0.30
(b) Clustering on methylation data
Height
0.00
0.10
0.20
0.30
