# Supplementary material for: Preferential Activation of the Hedgehog Pathway by Epigenetic Modulations in HPV Negative HNSCC Identified with Meta-Pathway Analysis
Source: PLoS One. 2013 Nov 4;8(11):e78127. doi: 10.1371/journal.pone.0078127 (PMC3817178; doi:10.1371/journal.pone.0078127)
Supplement: Methods S2 — Supplemental Methods. (PDF) [file pone.0078127.s010.pdf]

Preferential activation of the hedgehog pathway by epigenetic modulations in HPV negative HNSCC identified with meta-pathway analysis

Elana J. Fertig[1,*], Ana Markovic[1], Ludmila V. Danilova[1], Daria A. Gaykalova[2], Leslie Cope[1], Christine H. Chung[1], Michael F. Ochs[1,3], and Joseph A. Califano[1,2,4,*]

August 2, 2013

[1]*Department of Oncology, Sidney Kimmel Comprehensive Cancer Center, Johns Hopkins University, Baltimore, MD 21205;* [2]*Department of Otolaryngology-Head and Neck Surgery, Johns Hopkins Medical Institutions, Baltimore, MD 21205;* [3]*Department of Health Science Informatics, School of Medicine, Johns Hopkins University, Baltimore, MD 21205;* [4]*Milton J. Dance Head and Neck Center, Greater Baltimore Medical Center, Baltimore, MD 21204*

* authors to whom correspondence should be addressed

Supplemental Methods

CoGAPS matrix factorization model

CoGAPS decomposes a data matrix $\mathbf{D}$ with n rows and m samples into patterns (rows of $\mathbf{P}$) active to varying degrees represented in columns of the amplitude matrix $\mathbf{A}$ as

$$\mathbf{D} \sim \mathcal{N}\left(\mathbf{AP}, \Sigma\right), \tag{1}$$

where $\mathcal{N}$ represents a univariate normal distribution for the i, j entry of $\mathbf{D}$ with mean given by the corresponding entry of the matrix product $\mathbf{AP}$ and standard deviation in the i, j entry of Σ (Figure 1a). The CoGAPS algorithm [5] enforces sparsity and non-negativity of the elements of $\mathbf{A}$ and $\mathbf{P}$ to identify overlapping patterns active in the data. The number of rows of $\mathbf{A}$ and columns of $\mathbf{P}$ (p) represents the dimensionality of the factorization found based upon pattern robustness (described below). We note that the fit to the data is invariant to scale changes in $\mathbf{P}$ Because of the matrix multiplication in eq (1)

The non-zero values in columns of of **A** represent a collection of genes acting together in samples, determined from non-zero values in corresponding rows of **P**. Consistent with [7], we scale each inferred matrix **A** and **P** by the corresponding uncertainty estimated with the MCMC algorithm. As a result, values with small but certain non-zero values of **A** will have equivalent weight to large but uncertain non-zero values, thereby accounting for the vastly different scales of mRNA expression in different genes. We call the collection of inferred genes associated with each "pattern" meta-pathways, and the amplitude in the corresponding pattern the meta-pathway activity (Figure 1b). We note that in this application, both meta-pathways and their associated activity across samples are inferred from the genomic data alone, without prior biological knowledge of pathways or clinical/experimental annotations.

Error models for DNA methylation and gene expression data

We assumed a 10% uncertainty in each matrix element for the gene expression data based upon [6]. Assuming that methylation β values for each probe follow a β distribution [4], we approximated the standard deviation for $\tilde{\beta}$ as

$$\sigma_{\tilde{\beta}} = \left[\frac{1-\beta}{\beta\,(M+U+1)}\right]^{1/2}. \tag{2}$$

This error model is large for unmethylated genes ($\beta = 0$), basing the factorization strictly on the gene expression data reflecting variable reactivation of unmethylated genes. On the other hand, the error model of eq. (2) approaches zero when a gene is fully methylated ($\beta = 1$), driving the CoGAPS to find patterns which decay to zero for those samples and thus reflect epigenetic silencing of gene expression (Figure 2a). To reflect technical variability of the array, we set the minimum value of $\sigma_{\tilde{\beta}}$ to be the minimum standard deviation of 0.008 estimated from log transformed β values in replicate samples of Illumina Infinium HumanMethylation27 BeadChips arrays for a control cell line in the cancer genome atlas (TCGA) [3]. As a result, $\Sigma_{\tilde{\beta}}$ always remains above zero (Figure 2b), enabling CoGAPS to fit meta-pathways in the data without forcing a perfect fit to any datapoint

(Figure 2c).

Analysis of pattern robustness

Pattern association computed with ClutrFree [1, 2] is defined as the maximum Pearson correlation between each of the patterns inferred from CoGAPS for $p-1$ and each of the patterns for p. The relative thickness of lines in the tree generated from ClutrFree represents relative magnitude of this association [1]. Persistence quantifies the associations between each of patterns by comparing the genes that with statistically significant amplitudes in the **A** matrix for the patterns inferred from $p-1$ to statistically significant genes from each of the patterns at p [1, 2]. Both pattern association and persistence are used to assess the appropriate number of dimensions p [2, 7].

CoGAPS identified up to six robust patterns in HNSCC data

We applied CoGAPS [5] to the combined methylation and expression from HNSCC tumors and UPPP control sample. Identification of common expression and methylation patterns in these samples relies first on accurate identification of the number of patterns in the data matrix. In accordance with [2], we applied the factorization for p patterns ranging from two to fifteen and assessed the χ^2 fit and persistence in patterns (Figure 3). The χ^2 fit to the combined DNA methylation and expression dataset decayed monotonically with the number of patterns, with the sharpest rate of decline occurring between two and six patterns. The persistence of gene membership in patterns was constant when applying the matrix factorization for two to four patterns and then decayed gradually, and finally dropped sharply at eight patterns. We note that the persistence increased slightly at $p = 10$, suggesting greater similarity between CoGAPS patterns for $p = 10$ and $p = 9$ than $p = 8$ and $p = 9$. However, this robustness measure continued to decay beyond $p = 10$ and never regained the relative stability observed for p between two and four.

The correlation between patterns inferred across samples provided an additional metric for dimensionality selection. A tree diagram visualizing the relationships between these patterns (Fig-

ure 4(a)) showed that patterns identified in $p-1$ dimensions were also identified in the matrix factorization for p with a minimum correlation coefficient of 0.83 for up to six total patterns. Several of these patterns were not robustly identified (correlation coefficient below 0.5) once the total number of patterns increases beyond six. Taken together, these results suggest that the matrix factorization algorithm inferred robust solutions when the number of patterns is at most six. While the χ^2 fit and persistence implicate a single dimensionality in the datasets studied in [1, 7, 6], both metrics remained relatively stable for p between two and six in this HNSCC dataset. However, the relative drop in persistence at $p = 4$ and in χ^2 fit at $p = 6$ suggested our meta-pathway analysis decomposing the data matrix for p ranging from two to five.

Relative contribution of DNA methylation and gene expression to pattern stability

The agreement between the χ^2 fit between the methylation (Figure 4(b)) and relative degradation of the χ^2 fit compared to expression data alone (Figure 4(c)) further confirmed the dominant role of the methylation data in pattern inference. While the persistence likewise closely followed the persistence of the factorization for the methylation data alone (Figure 4(d)), the sharp decrease in persistence of the expression data (Figure 4(e)) was consistent with the decreased correlation between patterns occurring at p of six.

Although χ^2 error was larger for the integrated meta-pathway analysis than a similar analysis on gene expression data alone, both fits to the data represented a substantial improvement over the χ^2 error of 6×10^6 for the empty matrices used to initiate CoGAPS. Moreover, at $p = 5$ the fitted estimates for expression and methylation obtained from the product of $\mathbf{A}$ and $\mathbf{P}$ had greater anti-correlation for the combined dataset ($r = -0.28$) than did fitted estimates for expression alone with DNA methylation or the raw data ($r = -0.25$ for both). This anti-correlation was supported by use of the log transform of β, for which the corresponding error model (eq (2)) weighed highly methylated genes more in the decomposition (Figure 2b). Nonetheless, the accuracy of CoGAPS

fits to DNA methylation had comparable accuracy when genes were fully unmethylated suggesting little bias in the meta-pathways associated with DNA methylation changes (Figure 2c).

References

[1] G Bidaut and M F Ochs. ClutrFree: cluster tree visualization and interpretation. *Bioinformatics*, 20(16):2869–71, 2004.

[2] G Bidaut, K Suhre, J-M Claverie, and M F Ochs. Determination of strongly overlapping signaling activity from microarray data. *BMC Bioinformatics*, 7:99, 2006.

[3] Cancer Genome Atlas Research Network. Comprehensive genomic characterization defines human glioblastoma genes and core pathways. *Nature*, 455(7216):1061–8, 2008.

[4] P Du, X Zhang, C-C Huang, N Jafari, W A Kibbe, L Hou, and S M Lin. Comparison of beta-value and m-value methods for quantifying methylation levels by microarray analysis. *BMC Bioinformatics*, 11:587, 2010.

[5] E J Fertig, J Ding, A V Favorov, F Parmigiani, and M F Ochs. CoGAPS: an R/C++ package to identify patterns and biological process activity in transcriptomic data. *Bioinformatics*, 26(21):2792–3, 2010.

[6] E J Fertig, Q Ren, H Cheng, H Hatakeyama, A P Dicker, U Rodeck, M Considine, M F Ochs, and C H Chung. Gene expression signatures modulated by epidermal growth factor receptor activation and their relationship to cetuximab resistance in head and neck squamous cell carcinoma. *BMC Genomics*, 13(1):160, 2012.

[7] M F Ochs, L Rink, C Tarn, S Mburu, T Taguchi, B Eisenberg, and A K Godwin. Detection of treatment-induced changes in signaling pathways in gastrointestinal stromal tumors using transcriptomic data. *Cancer Res*, 69(23):9125–32, 2009.

Figures

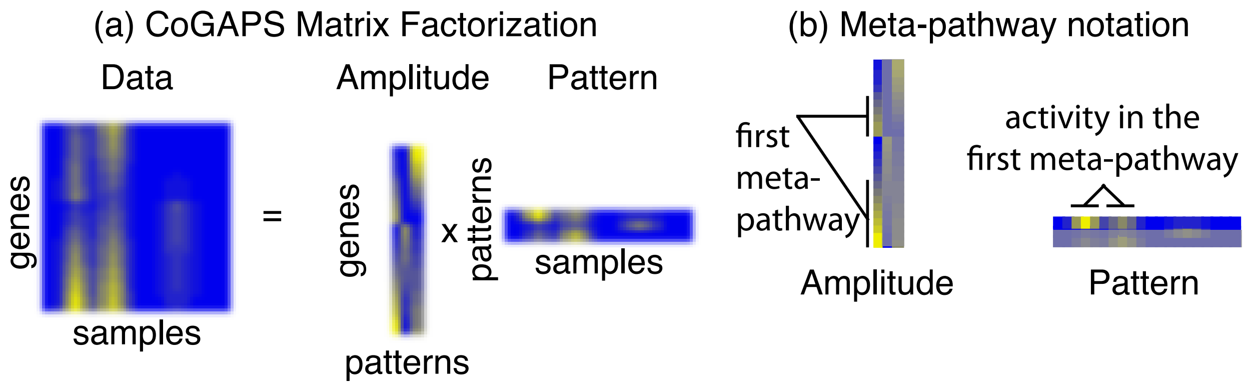

Figure 1: (a) Illustration of CoGAPS matrix decomposition from eq (1) and (b) associated inference of gene membership in meta-pathways and activity in samples.

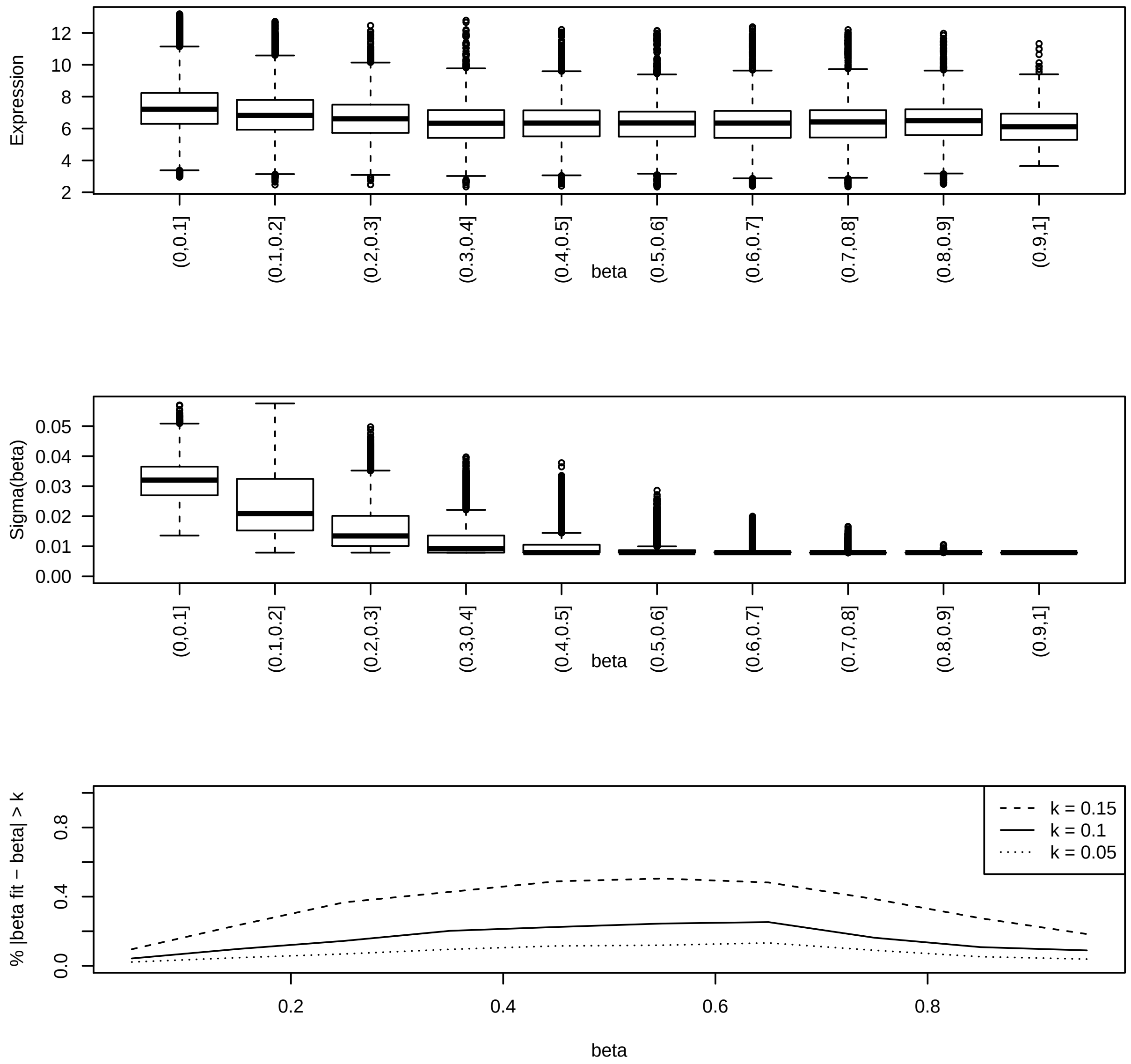

Figure 2: (a) Boxplot of gene expression for genes whose DNA methylation is within the indicated range, reflecting hypothesized anti-correlation between gene expression and DNA methylation ($r = -0.25$). (b) Boxplot of estimated uncertainty for DNA methylation from eq (2) for DNA methylation values within each indicated range. (c) Percentage of DNA methylation measurements for which the CoGAPS fit to β disagrees from the measured value by more than a threshold k for each measured value of β.

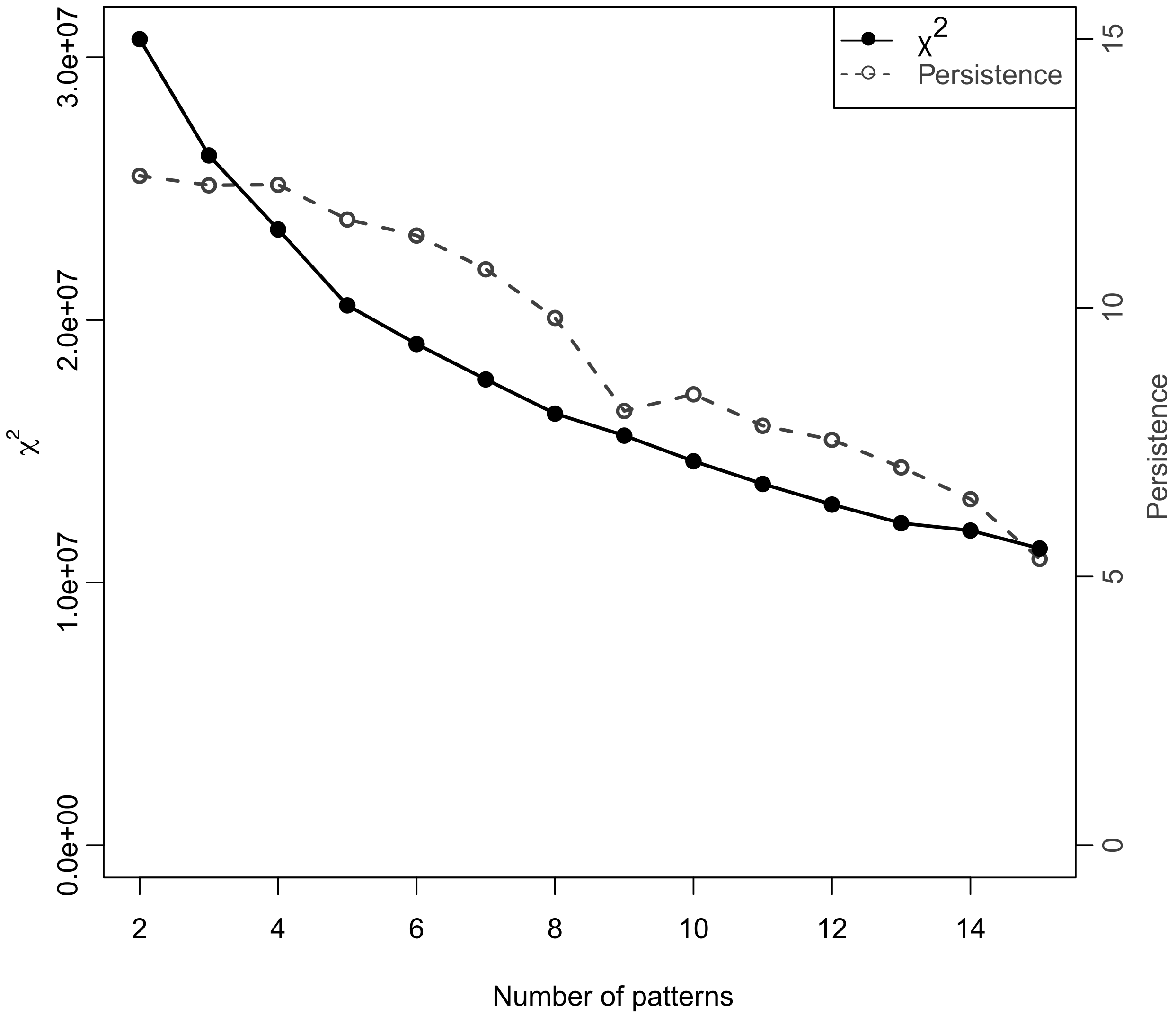

Figure 3: χ^2 of fit to data (left axis; black) and persistence of genes in patterns (right axis; grey) for matrix factorizations for total number of patterns p ranging from 2 to 15.

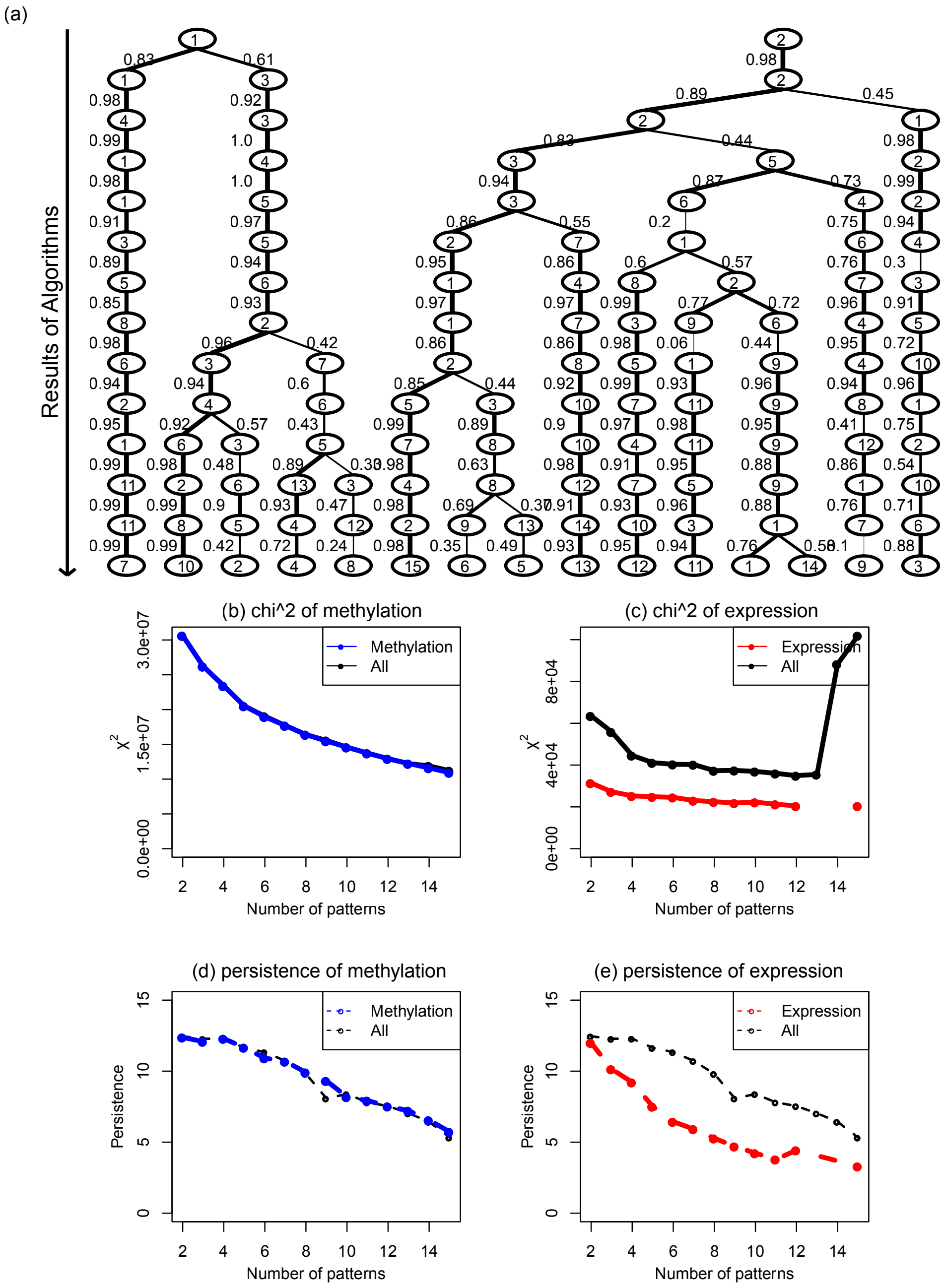

Figure 4: (a) Tree diagram generated with ClutrFree [1] showing the relationship between patterns inferred from applying the CoGAPS [5] matrix factorization to combined gene expression data and log transformed DNA methylation data for total number of patterns p from 2 to 15 (from top to bottom). Thickness of lines and numbers represent the Pearson correlation between each of the subsequent patterns identified in the matrix factorization. (b) Comparison of χ^2 fit as a function of the number of patterns when applying the matrix factorization to only the log transformed DNA methylation data (blue) or to the combined data calculated only for DNA methylation genes (black). (c) As in (b) with expression data (red). (d) Comparison of the persistence between patterns computed with ClutrFree [1] for log transformed DNA methylation data only (blue) and combined methylation and expression data (black). (e) As for (d) with expression data (red).
